# Dihydromyricetin Alleviates Diabetic Neuropathic Pain and Depression Comorbidity Symptoms by Inhibiting P2X_7_ Receptor

**DOI:** 10.3389/fpsyt.2019.00770

**Published:** 2019-10-18

**Authors:** Shu Guan, Yulin Shen, Huixiang Ge, Wei Xiong, Lingkun He, Lijuan Liu, Cancan Yin, Xingyu Wei, Yun Gao

**Affiliations:** ^1^Department of Physiology, Basic Medical College, Nanchang University, Nanchang, China; ^2^Sport Biological Centre, China Institute of Sport Science, Beijing, China; ^3^Department of Preventive Dentistry, Affiliated Stomatological Hospital of Nanchang University, Nanchang, China; ^4^Basic Medical College of Grade 2017, Nanchang University, Nanchang, China; ^5^Jiangxi Provincial Key Laboratory of Autonomic Nervous Function and Disease, Nanchang University, Nanchang, China

**Keywords:** dihydromyricetin, P2X_7_ receptor, diabetic neuropathic pain, major depressive disorder, dorsal root ganglion, spinal cord, hippocampus

## Abstract

Diabetic neuropathic pain (DNP) and major depressive disorder (MDD) are common complications of diabetes mellitus and mutually affect each other. As a member of the ATP-gated ion channel family, P2X_7_ receptor is associated with the transduction of pain signal and the onset of depression. The aim of this study was to investigate the effects of dihydromyricetin (DHM) on rats with comorbid DNP and MDD. After the comorbid model was established, rat behavior changes were monitored by measuring the mechanical withdrawal threshold, thermal withdrawal latency, sugar water preference, immobility time in the forced-swim test, and open-field test parameters. The expressions of P2X_7_ receptor in the dorsal root ganglia (DRGs), spinal cord, and hippocampus were assessed by quantitative real-time PCR, Western blotting, and double immunofluorescence. We found that hyperalgesia, allodynia, and depressive behaviors of rats with comorbid DNP and MDD were relieved by treatment with DHM or application of a short-hairpin RNA for P2X_7_ receptor. The expression levels of P2X_7_, phosphorylated extracellular signal–regulated kinase 1/2, tumor necrosis factor α, and interleukin 1ß were increased in the DRGs, spinal cord, and hippocampus of rats in the model group but restored after DHM or P2X_7_ short-hairpin RNA treatment. In conclusion, P2X_7_ receptor in the DRGs, spinal cord, and hippocampus participates in the transduction of DNP and MDD signals. DHM seems to relieve comorbid DNP and MDD by reducing the expression of P2X_7_ receptor in the DRGs, spinal cord, and hippocampus and may be an effective new drug for the treatment of patients with both DNP and MDD.

## Introduction

Given the high prevalence of diabetes mellitus worldwide, diabetic neuropathic pain (DNP) has become a relatively common condition (1, 2). DNP often involves primary injury or dysfunction of the peripheral or central nervous system (3, 4). Although DNP is one of the main symptoms of diabetic neuropathy, its pathophysiological mechanisms are not yet fully understood (5).

Depression, also known as major depressive disorder (MDD), is a serious medical condition affecting public health. Mild and severe depression is closely associated with increased mortality rates in patients with diabetes mellitus (6). Painful diabetic polyneuropathy is a greater determinant of depression than other diabetes-related complications and comorbidities (7), and depression severity depends on the intensity of pain (8). Comorbid DNP and MDD seriously affect the quality of life and are more difficult to treat than either DNP or MDD in isolation. At present, the relevant mechanisms have not been thoroughly elucidated and require further research.

After nerve injury and inflammation, nerve endings release large amounts of adenosine triphosphate, an important neurotransmitter that activates several purinergic receptors and is necessary for many biological and pathological functions (9, 10). P2X_7_ receptor is a ligand-gated nonselective cation channel receptor (11–13) that is closely associated with neuropathic pain and depression (14, 15). Many studies have confirmed that P2X_7_ receptor activation is involved in depression progression (16–18). Our previous experiments confirmed that hippocampal P2X_7_ receptor expression was noticeably higher in rats with comorbid DNP and MDD than in control rats (19). We speculate that P2X_7_ receptor might be a common target for the two comorbid diseases. However, the effects of comorbid DNP and MDD on P2X_7_ receptor expression in the dorsal root ganglia (DRGs) and spinal cord have not been reported.

**Figure 1:**
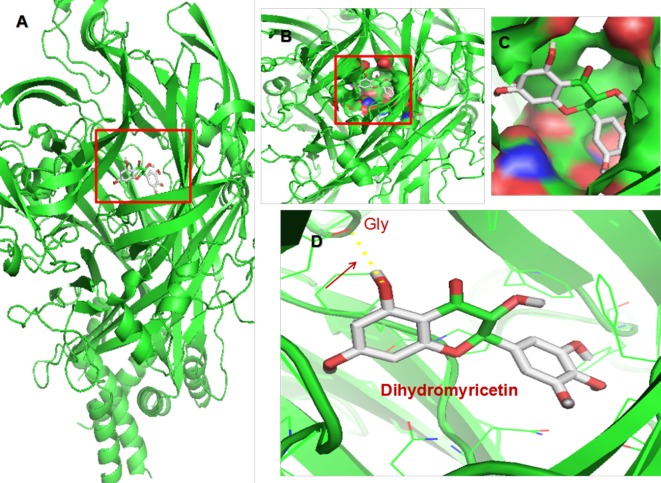
Molecular docking of dihydromyricetin (DHM) on P2X_7_ receptor. **(A)** Simulation modeling of DHM docking on P2X_7_ receptor was performed by a computer. Molecular docking prediction of DHM on P2X_7_ receptor was performed by AutoDock 4.2. **(B**–**D)** Enlarged view indicating the perfect match enabling DHM to interact with P2X_7_ receptor.

The flavonoid dihydromyricetin (DHM) is the most abundant organic chemical in vine tea, which is made from *Ampelopsis grossedentata*, and provides myriad health benefits, including anti-inflammatory, antitumor, and rapid antidepressant-like effects (20, 21). Homology modeling and molecular docking analysis, which predicts ligand binding at a protein’s active sites (22), suggested that DHM is capable of high-affinity binding to P2X_7_ receptor (**Table 1** and **Figure 1**). Thus, we hypothesized that DHM could be used to treat the comorbid symptoms by acting on P2X_7_ receptor. The aim of this study was to investigate whether DHM treatment can alleviate comorbid DNP and MDD by inhibiting P2X_7_ receptor expression in the DRGs, spinal cord, and hippocampus.

**Table 1:** MOE (Molecular Operating Environment, a docking software) score of P2X_7_ receptor docking and dihydromyricetin (kcal/mol).

Mode	Affinity	Dist from best mode	
	(kcal/mol)	RMSD lb	RMSD ub
1	-7.4	0	0
2	-7.2	1.600	2.344
3	-7.1	16.017	18.330
4	-7.1	4.546	6.709
5	-7.0	16.775	19.643
6	-7.0	16.089	18.520
7	-6.9	62.551	65.298
8	-6.9	18.809	22.016
9	-6.8	21.204	23.029

The predicted binding affinity is in kcal/mol (energy). *rmsd: RMSD values are calculated relative to the best mode and use only movable heavy atoms. Two variants of RMSD metrics are provided, rmsd/lb (RMSD lower bound: matches each atom in one conformation with itself in the other conformation, ignoring any symmetry) and rmsd/ub (RMSD upper bound: rmsd/lb[c1, c2] = max[rmsd*′*{c1, c2}, rmsd*′*{c2, c1}], and rmsd*′* matches each atom in one conformation with the closest atom of the same element type in the other conformation), differing in how the atoms are matched in the distance calculation. There is a strong reaction between ligand and protein when the binding affinity is bigger than −6.0 kcal/mol. The rmsd/lb and rmsd/ub of modes 1 to 5, as well as of modes 6 to 8 are much similar, which indicates that those modes are located in one docking pocket. In summary, molecular docking of dihydromyricetin on a mode-h protein P2X_7_ is stable.

## Materials and Methods

### Animals and Treatments

**Figure 2:**
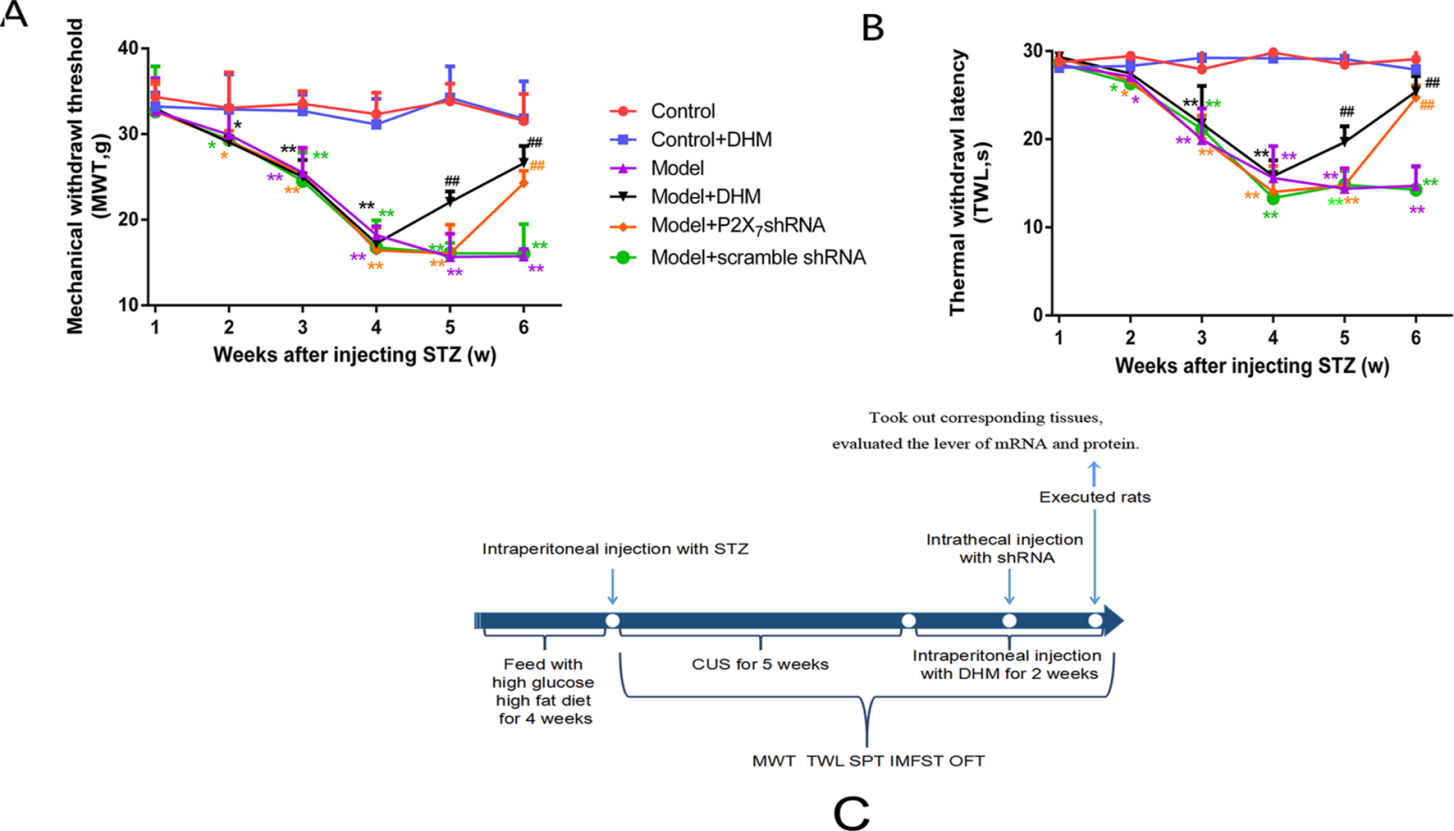
Effects of dihydromyricetin (DHM) on mechanical withdrawal threshold (MWT; **A**) and thermal withdrawal latency (TWL; **B**) values in rats with diabetic neuropathic pain and major depressive disorder (model). **(C)** The timeline of treatments used in this study. Data are displayed as means ± standard errors of the means. *p < 0.05, **p < 0.01 vs. control group; ^##^ *p* < 0.01 vs. model group.

Male Sprague–Dawley rats (180–220 g) were provided by the Centre of Laboratory Animal Science of Nanchang University. The procedures of this study were approved by the Animal Care and Use Committee of Nanchang University Medical School and were performed according to IASP(International Association for the Study Pain)’s ethical guidelines for pain research in animals. Rats were housed under controlled conditions at 25°C temperature and 60% humidity, with freely available food and water. Five rats were housed in each cage. The timeline of this study is shown in **Figure 2C**.

#### Generation of DNP and MDD Rat Model

During the week before the start of the experiment, rats were fed a normal diet. After that, they were fed high-glucose, high-fat diet for 4 weeks. After the end of the 4 weeks, rats were starved for more than 12 h and were then given an intraperitoneal (i.p.) injection of streptozotocin (STZ; 35 mg/kg). Blood glucose was measured after food consumption. Rats whose blood glucose levels were higher than 16.7 mmol/l were chosen as having type 2 diabetes mellitus. For the next 5 weeks after injecting STZ, chronic unpredictable stress (CUS) stimuli were given randomly. Meanwhile, we measured responses in several behavioral tests once a week to verify that rats had both DNP and MDD, as follows: thermal withdrawal and mechanical withdrawal tests, sucrose preference (SP) test, forced-swimming test (FST), and open-field test (OFT). The CUS stimuli included food deprivation (24 h), cold swimming (4°C, 5 min), water deprivation (24 h), heat stress (45°C, 5 min), reverse light/dark cycle, no stressor, and clip of the tail (1 min) (13). Rats were exposed to one of the seven daily stressors randomly for 5 weeks.

#### Treatments

Seventy-two male rats were randomly divided into six groups: (1) control, (2) control + DHM, (3) comorbid DNP and MDD model (model), (4) DHM treatment group (model + DHM), (5) P2X_7_ receptor short-hairpin RNA (shRNA) treatment group (model + P2X_7_ shRNA), and (6) scramble shRNA treatment group (model + scramble shRNA). Rats in the control + DHM and model + DHM groups were treated with DHM *via* i.p. injection once a day, at a dose of 30 mg/kg, for 14 consecutive days.

The transfection complex consisting of shRNA (P2X_7_ or scramble shRNA) and transfection reagent at a ratio of 1:2 (μg/μl) was prepared using the Entranster™ *in vivo* transfection reagent (Engreen Biosystem Company of Beijing), according to the manufacturer’s instructions. The complex was intrathecally injected into rats of the model + P2X_7_ and model + scramble shRNA groups. The behavior of rats in the two groups was assessed once a day after the intrathecal injection. The sequences of P2X_7_ shRNA were as follows: ′-CACCGTGCAGTGAATGAGTACTACGAATAGTACTCATTCACTGCAC-3′ and 3′-CACGTCACTTACTCATGATGCTTATCATGAGTAAGTGACGTGAAAA-5′.

DHM was purchased from Zelang, Nanjing, China. P2X_7_ shRNA and scramble shRNA were synthesized by Novobio, Shanghai, China.

### Mechanical Withdrawal Test

Mechanical withdrawal threshold (MWT) was evaluated by observing the withdrawal responses to mechanical stimulation, induced by using the BME-404 electronic mechanical stimulator (provided by the Institute of Biomedical Engineering of Chinese Academy of Medical Sciences). The end-face diameter of the test needle, the pressure measurement range, and pressure measurement resolution of the stimulator were 0.6 mm, 0.1 to 50 g, and 0.05 g, respectively. Before evaluation, each rat was placed in a clean glass box positioned on the sieve of a metal frame for an adaptive period of at least 30 min. The test needle touched the place between the third and fourth metatarsus of the left hind paws, until the rat attempted to withdraw its paw. The computer recorded the pressure values automatically. The stimulus alternated between the left and right hind paws at 5-min intervals. The MWT was calculated as the mean of three consecutive stable values, expressed in grams, and was determined by one observer.

### Thermal Withdrawal Test

Thermal withdrawal latency (TWL) was evaluated by measuring the latency to hind paw withdrawal from a thermal stimulus, administered using the BME-410C Thermal Paw Stimulation System (provided by the Institute of Biomedical Engineering of Chinese Academy of Medical Sciences). Before evaluation, each rat was placed on a glass plate in a transparent, square, bottomless acrylic box, for an adaptive period of at least 30 min. A beam of radiant heat was oriented at the plantar surface of the rat’s paws. The activation of the beam simultaneously activated a timer. The cutoff time for heat stimulation was 30 s. The light beam was switched off when the animal lifted its paw, and the timing was over. The time on the screen of the apparatus was designated as the TWL and was expressed in seconds. The hind paw withdrawal was tested in triplicate, and hind paws were alternated at 5-min intervals.

### Sucrose Preference Test

Before the test, rats were fasted for 24 h and then placed individually in separate cages. Two identical water bottles, one containing 100 ml of 1% sucrose in water and another containing 100 ml of pure water, were placed in every cage at the same time. SP was evaluated by measuring the levels of sugar–water and pure-water consumption in 1 hour. The SP rate was calculated as the ratio of sugar water/total liquid consumption × 100%. This test reflects a lack of pleasure.

### Forced Swimming Test

Rats were placed in an 80-cm-high glass cylinder with a 40-cm inner diameter. Water temperature was approximately 20, and water depth was 30 cm. The immobility time (IT) of rats in the water, i.e., when rats stopped struggling and floated in a fixed shape, and the swimming time of each rat were recorded for 5 min and expressed in seconds.

### Open-Field Test

Before the test, rats were placed in the dark for 30 min to adapt to the environment. Then, they were placed in a black box that measured 40 × 60 × 50 cm. Each rat was placed gently in the middle of the box, and the distance navigated by the animal was recorded using a Canon Powershot A610 camera (Canon Co. local distributer, Tehran, Iran) during a 5-min session. The recorded videos were analyzed and processed using MATLAB (MathWorks Co., Natick, MA, USA) to determine the total distance traveled, expressed in centimeters. The apparatus was cleaned with a 10% ethanol solution before the next animal was introduced into the box.

### Quantitative Real-Time PCR

Rats were anesthetized by i.p. injection of 10% chloral hydrate (batch no. 050101; Shanghai Xingya Medical Company, China). DRGs, the spinal cord at the level of L4–L5 vertebrae, and hippocampus were isolated immediately after sacrifice from rats in different groups, flushed with ice-cold phosphate-buffered saline (PBS), and stored in RNA Store solution at −20°C until further use. All instruments were treated with DEPC before use.

Total RNA was separately isolated from DRGs, spinal cord, and hippocampus using the TRIzol Total RNA Reagent (Beijing TransGen Biotech Co.). Complementary DNA synthesis was performed with 2 µg total RNA using the RevertAid™ HMinus First Strand cDNA Synthesis Kit. The primers were designed using Primer Express 3.0 Software (Applied Biosystems), and sequences were as follows: β-actin forward 5′-TAAAGACCTCTATGCCAACA -3′ and reverse 3′-CACGATGGAGGGGCCGGACTCATC-5′; P2X_7_ forward 5′-GATGGATGGACCCACAAAGT-3′ and reverse 3′-GCTTCTTTCCCTTCCTCAGC-5′.

Quantitative real-time PCR was performed using the SYBR® Green Master Mix in the ABI PRISM® 7500 Sequence Detection System (Applied Biosystems Inc., Foster City, CA). The expression of each gene was quantified using the ΔΔCT method, with CT as the threshold cycle. The relative levels of target genes normalized to the sample with the lowest CT are presented as 2^−ΔΔCT^.

### Western Blotting

After rats were anesthetized, DRGs, spinal cord at L4–L5, and hippocampus were separated and flushed with ice-cold PBS. Tissues were positioned in the spherical part of a 2-ml homogenizer and homogenized in RIPA lysis buffer (50 mM Tris-Cl, pH 8.0, 150 mM NaCl, 0.1% sodium dodecyl sulfate, 1% Nonidet P-40, 0.02% sodium deoxycholate, 100 mg/ml phenylmethylsulfonyl fluoride, and 1 mg/ml aprotinin) containing protease inhibitors. Tissues were ground for 30 min on ice and centrifuged at 4°C at 12,000 rpm for 10 min. The supernatants were collected, diluted with 6× loading buffer, and heated to 95 for 10 min. The protein concentration was calculated with the BCA Protein Assay Kit, and samples were kept at −20°C until use. Proteins in samples from each group (20 μg) were separated by 12% sodium dodecyl sulfate–polyacrylamide gel electrophoresis, using Bio-Rad electrophoresis device, and transferred onto polyvinylidene fluoride membranes. Polyvinylidene fluoride membranes were blocked with 5% nonfat dry milk in 1× TBST (Tris-Buffered Saline and Tween 20) for 2 h at room temperature, followed by incubation with antibodies against P2X_7_ (1:500; Alomone Labs, Jerusalem, Israel), β-actin (1:1,000; Beijing Zhongshan Biotech Co., China), extracellular signal–regulated kinases 1/2 (ERK1/2; 1:1,000; Cell Signaling Technology Inc, Boston, MA, USA), and phosphorylated (p)-ERK1/2 (1:1,000; Cell Signaling) at 4°C overnight. Membranes were washed three times with 1× TBST, 10 min each, incubated for 2 h at room temperature with horseradish peroxidase–conjugated secondary goat anti-rabbit immunoglobulin G and goat anti-mouse immunoglobulin G antibodies (1:2000; Beijing Zhongshan Biotech Co., China) in blocking buffer, and washed again three times with 1× TBST, 10 min each. Labeled proteins were then visualized with enhanced chemiluminescence on a Bio-Rad system. Band intensities were quantified using Image-Pro Plus software, and the intensities of target proteins were normalized against the respective β-actin internal control.

### Double-Immunofluorescence Labeling

Rats were anesthetized with 10% chloral hydrate and were transcardially perfused with 4% paraformaldehyde (PFA). The DRGs, spinal cord at L4–L5, and hippocampus were removed and fixed in 4% PFA at 4°C for 2 h at room temperature. The tissues were immersed in 30% sucrose solution (in 4% PFA) for 24 h, at 4°C, for dehydration; solutions were changed every 8 h. Tissues were cut into 12- or 8-μm-thick slices in a cryostat (Leica). The sections were placed at 37°C for 2 h and then stored at −20°C until use.

Before staining, sections were balanced at room temperature, rinsed with 0.01 M PBS for 5 min × three times, incubated with 0.3% Triton X-100, and washed again with PBS for 5 min × three times. Then, slices were incubated in 10% goat serum for 1 h at 37°C, followed by incubation with the diluted antibodies (rabbit anti-P2X_7,_ 1:200, Alomone Labs; and mouse anti–glial fibrillary acidic protein [GFAP], 1:200, Millipore) overnight at 4°C. The next day, sections were placed at room temperature for 30 min and washed with PBS (three × 5 min); secondary antibodies (goat anti-mouse 1:800 and goat anti-rabbit 1:800; Abcam, USA) were added on sections at 37°C for 1 h. Sections were washed for 5 min × three times with PBS, sealed with antifade solution, and imaged using a fluorescence microscope (Olympus, Tokyo, Japan). Image-Pro Plus6.0 software was used to analyze the immunofluorescence intensity ratio of P2X_7_ and GFAP coexpression normalized to control values.

### Enzyme-Linked Immunosorbent Assay

For assessing the levels of tumor necrosis factor α (TNF-α) and interleukin 1β (IL-1β), we performed enzyme-linked immunosorbent assay (ELISA) using Rat PicoKine™ ELISA Kit (Boster Biological Technology), according to the manufacturer’s instructions. Experimental samples (diluted to 100 µl) or control were loaded in triplicates, and 100 µl of antibody solution (1× biotinylated anti-rat TNF-α or IL-1β antibody) was added to each well and incubated for 60 min at 37°C. After washing three times with 1× wash buffer, 100 µl of 1× avidin–biotin–peroxidase complex was added to each well and incubated for 30 min at 37°C. Wells were washed five times with 1× wash buffer, and 90 µl of color developing reagent was added to each well and incubated in the dark for 25 min at 37°C. The reaction was stopped by adding 100 µl of stop solution to each well. The absorbance at 450 nm was read using a microplate reader.

### Statistical Analysis

Statistical analyses were performed using SPSS 21.0 software. Data were analyzed by one-way analysis of variance. The experimental results are expressed as mean ± standard error of the mean and were considered significant at *p* < 0.05.

## Results

### Effect of DHM on MWT and TWL in Rats With Comorbid DNP and MDD

We generated a comorbid symptom rat model by injecting rats with STZ and subjecting them to CUS stimulation for 5 weeks. After 5 weeks, both MWT and TWL were significantly lower in the model group than in the control group (*p*< 0.05), thus confirming the efficiency of our model. Two weeks after injection of DHM or 1 week after injection of P2X_7_ shRNA, the MWT and TWL were significantly lower in the model + P2X_7_ shRNA and model + DHM groups than in the model group (*p* < 0.05), indicating that both P2X_7_ shRNA and DHM treatment relieve neuropathic pain–related behavior in rats with DNP and MDD (**Figures 2A, B**).

### Effect of DHM on SP, Distance in the OFT, and IT in Rats With Comorbid DNP and MDD

**Figure 3:**
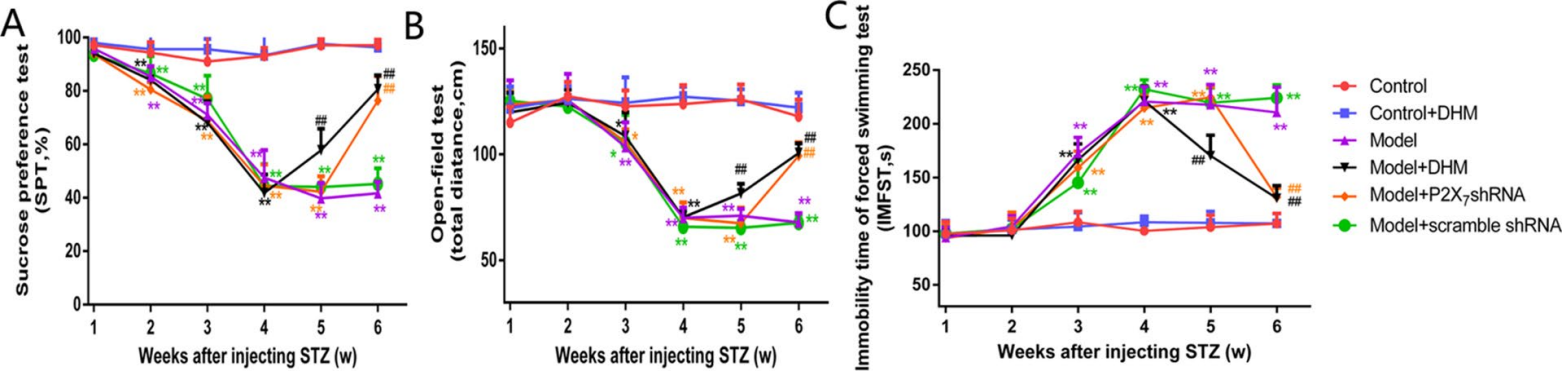
Effects of dihydromyricetin (DHM) on sucrose preference (SP; **A**), distance traveled in the open-field test (OFT; **B**), and immobility time (IT) in the forced-swimming test **(C)** in rats with diabetic neuropathic pain (DNP) and major depressive disorder (MDD) (model). Data are displayed as the means ± standard error of the mean. *p < 0.05,**p < 0.01 vs. control group; ^##^ *p* < 0.01 vs. model group.

Our analysis of SP in the respective test and of the distance traveled in the OFT showed that both values were markedly lower in the model than in the control group; in contrast, the IT in the FST was obviously higher in the model than in the control group, further confirming the successful generation of the DNP/MDD rat model (*p* < 0.05). Two weeks after injection with DHM or 1 week after injection with P2X_7_ receptor shRNA, the values for SP and OFT distance were significantly lower in the model + P2X_7_ shRNA and model + DHM groups than in the model group (*p* < 0.05). In addition, the IT in the FST was shorter in the model + P2X_7_ shRNA and model + DHM groups than in the model group (*p* < 0.05). The above results indicate that treatment with DHM or P2X_7_ shRNA relieves depression-like behaviors in model rats. Simultaneously, anhedonia improved, as evidenced by the higher values in the SPT and OFT and by the reduced IT in the FST in rats with DNP and MDD, indicating that DHM or P2X_7_ shRNA may moderate depressive symptoms (**Figure 3**).

### Effects of DHM on the Expression Levels of P2X_7_ Receptor in DRGs, Spinal Cord, and Hippocampus of Rats With Comorbid DNP and MDD

**Figure 4:**
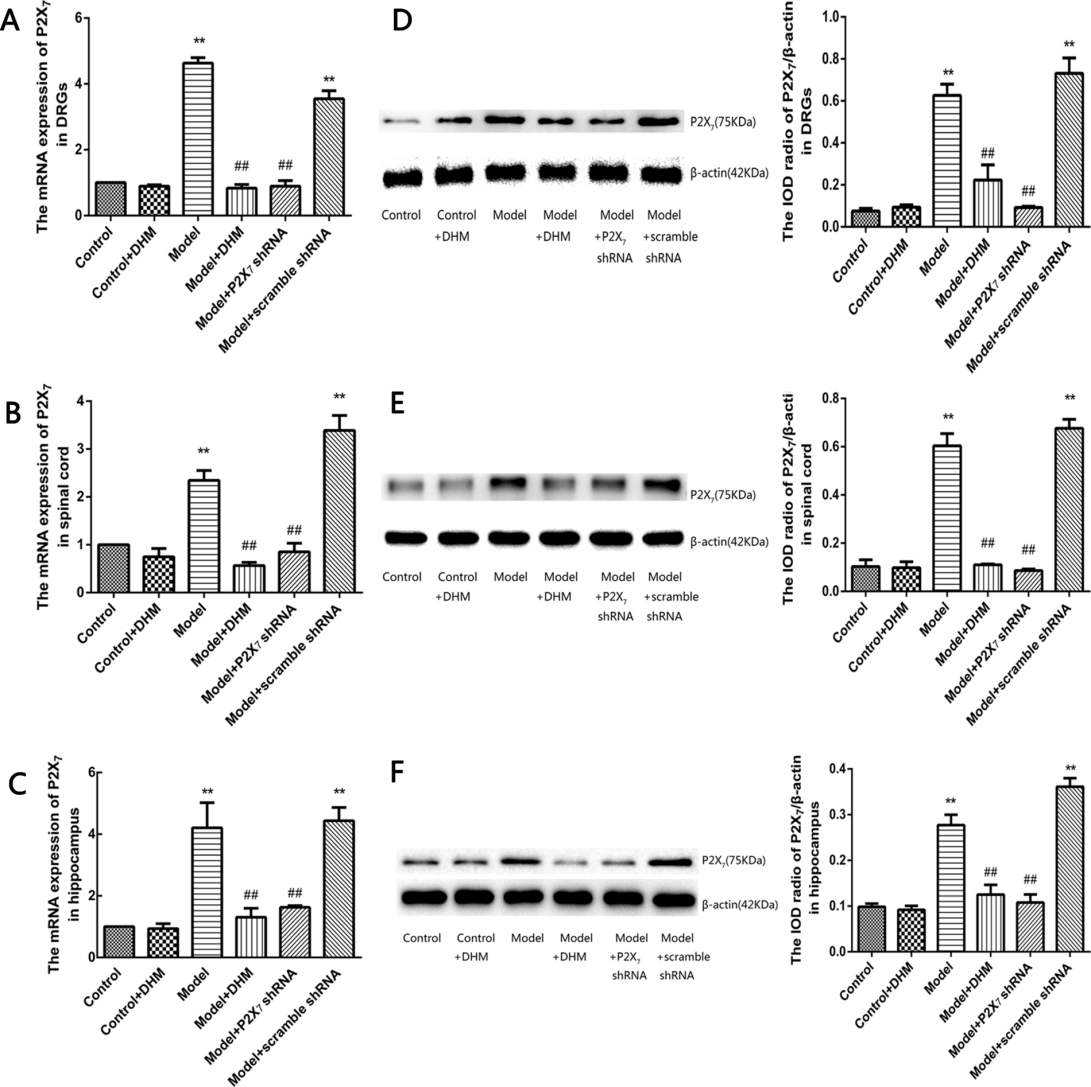
Effects of dihydromyricetin (DHM) on the expression of P2X_7_ mRNA in the dorsal root ganglia (DRGs; **A**), spinal cord **(B)**, and hippocampus **(C)** of rats with diabetic neuropathic pain (DNP) and major depressive disorder (MDD) (model). Effects of DHM on the expression of P2X_7_ protein in the dorsal root ganglia (DRGs; **D**), spinal cord **(E)**, and hippocampus **(F)** of rats with model. Data are displayed as the means ± standard error of the mean. **p<0.01 vs. control group; ^##^ *p* < 0.01 vs. model group.

The expression levels of P2X_7_ mRNA and protein were examined in the DRGs, spinal cord, and hippocampus in rats from each group by quantitative real-time PCR and Western blotting (**Figure 4**), respectively. In all tissues, the levels of P2X_7_ mRNA and protein were higher in rats in the model than in the control group. However, treatment with DHM or P2X_7_ shRNA significantly decreased the expression of P2X_7_ mRNA and protein (*p* < 0.05).

**Figure 5:**
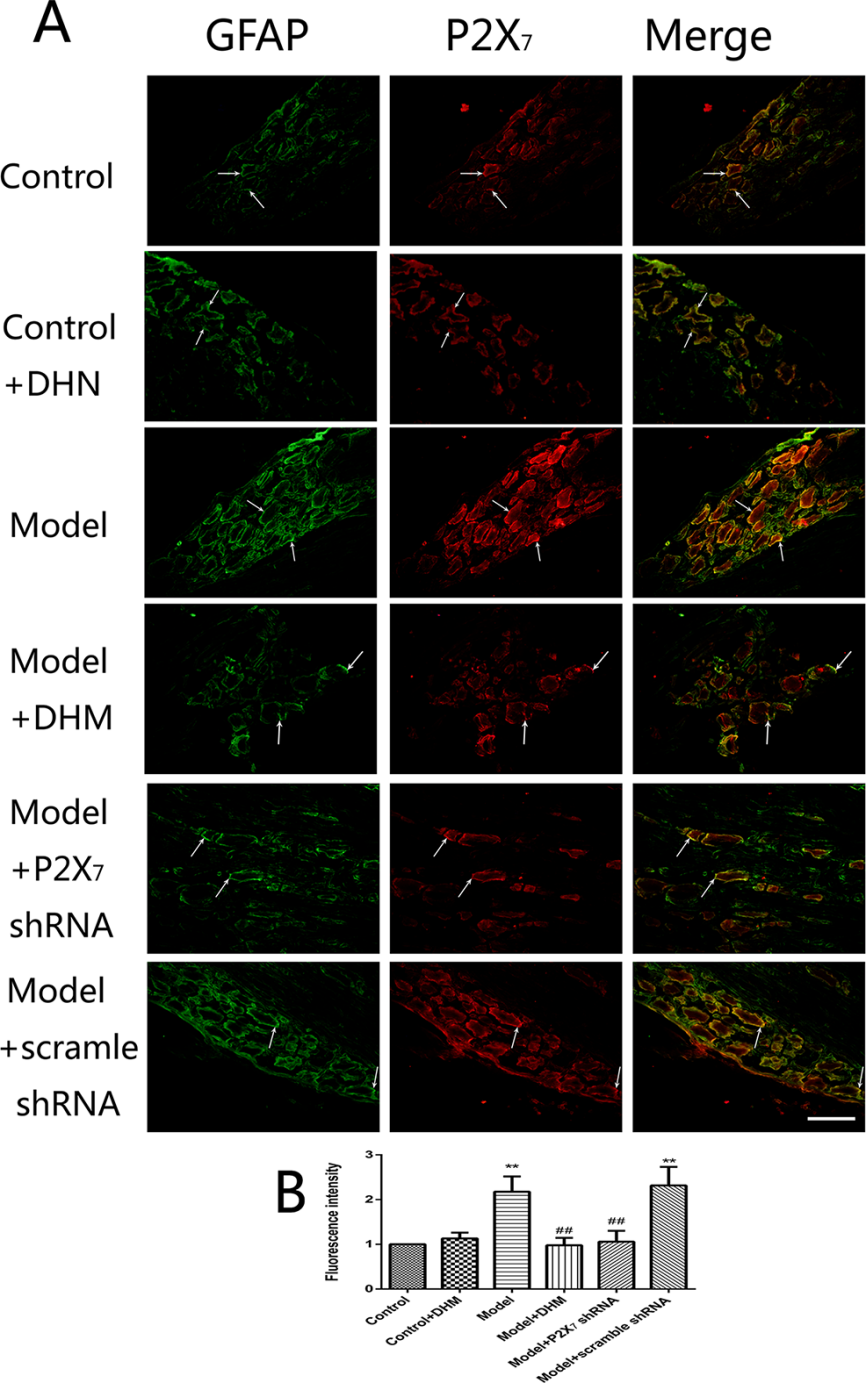
Effects of dihydromyricetin (DHM) on the coexpression of P2X_7_ and glial fibrillary acidic protein (GFAP) in the dorsal root ganglia (DRGs) of rats with diabetic neuropathic pain (DNP) and major depressive disorder (MDD) (model). Data are displayed as the means ± standard error of the mean. **p < 0.01 vs. control group; ^##^ *p* < 0.01 vs. model group. Scale bars, 100 µm.

**Figure 6:**
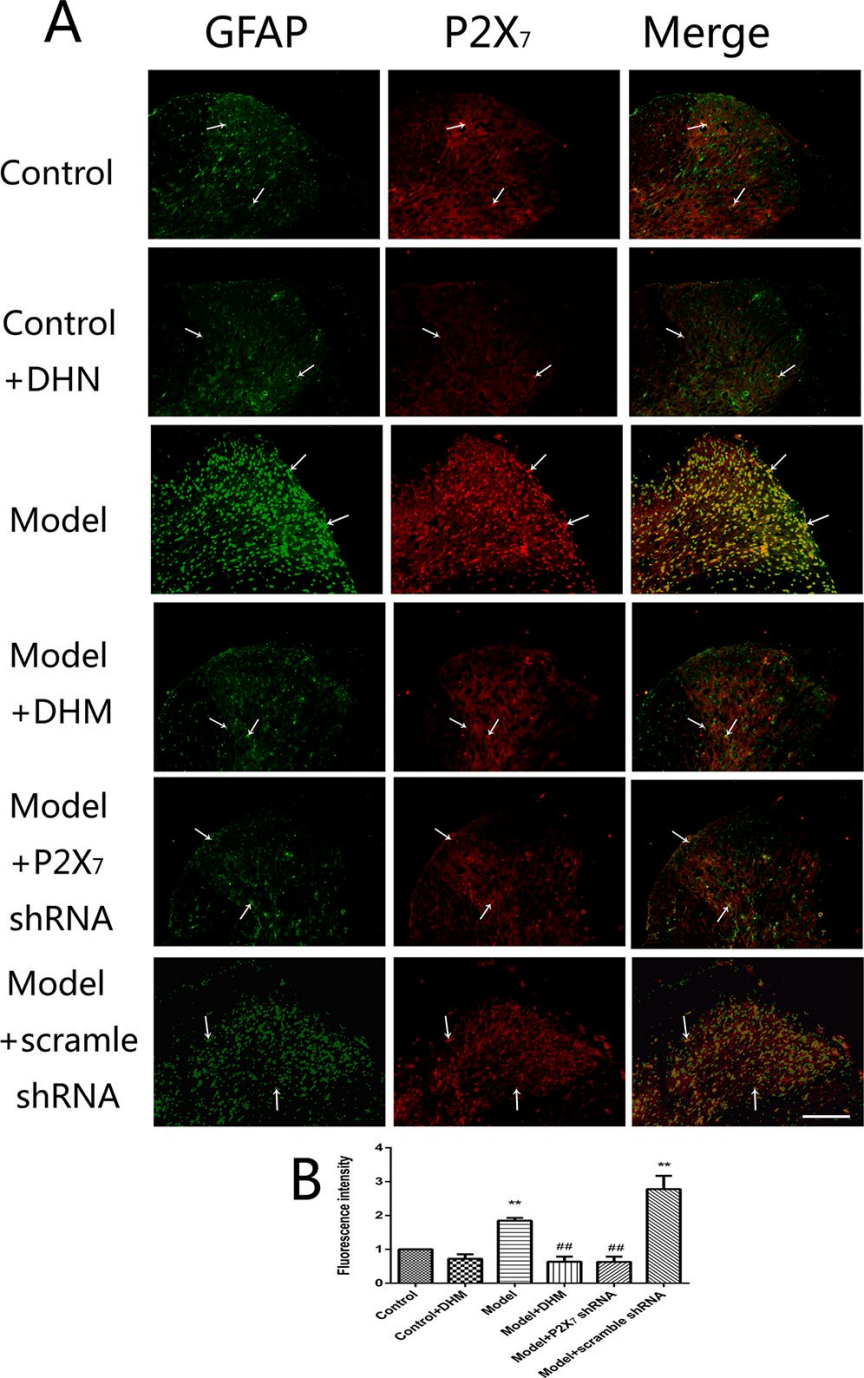
Effects of dihydromyricetin (DHM) on the coexpression of P2X_7_ and glial fibrillary acidic protein (GFAP) in the spinal cord of rats with diabetic neuropathic pain (DNP) and major depressive disorder (MDD) (model). Data are displayed as the means ± standard error of the mean. **p < 0.01 vs. control group; ^##^ *p* < 0.01 vs. model group. Scale bars, 100 µm.

**Figure 7:**
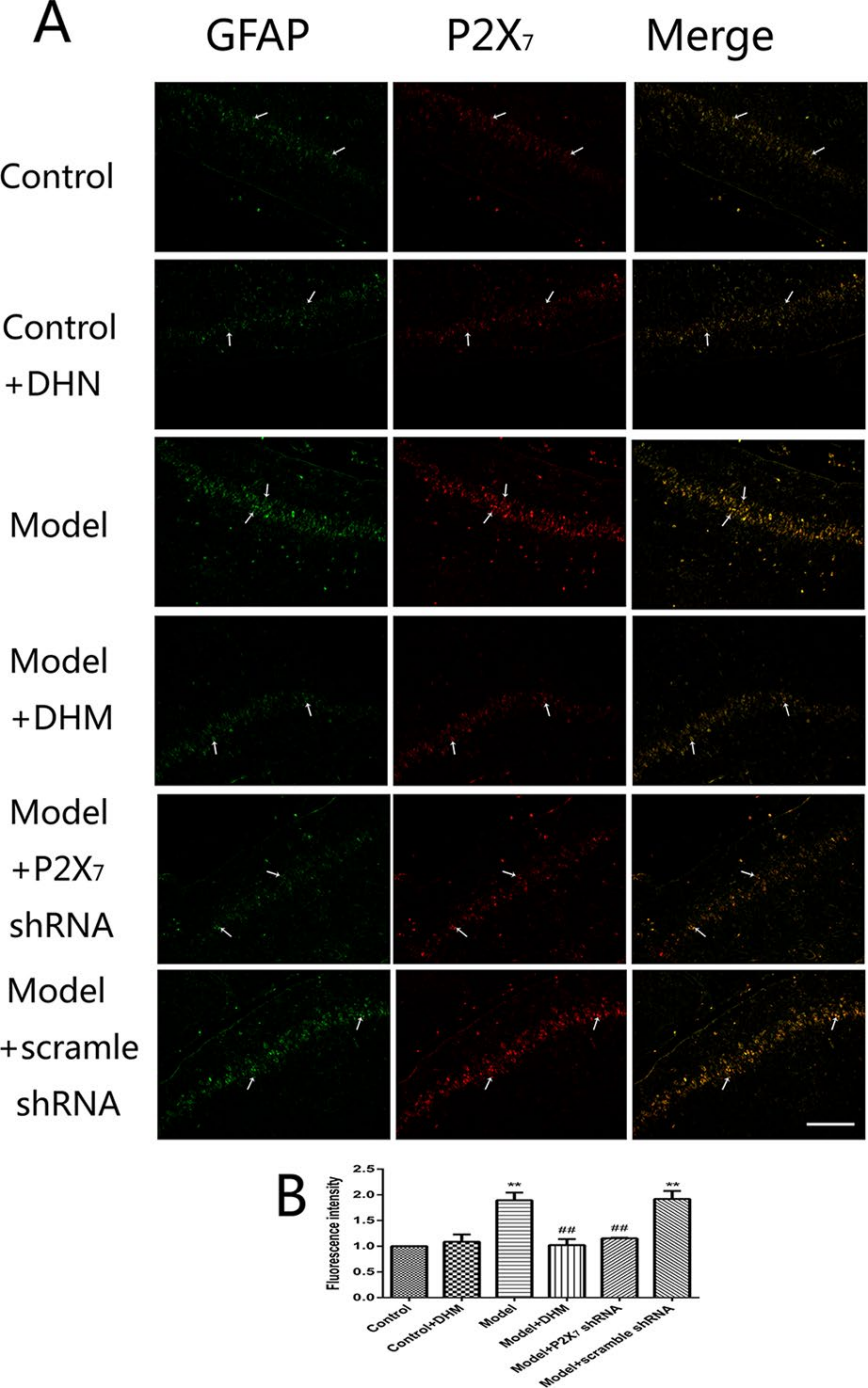
Effects of dihydromyricetin (DHM) on the coexpression of P2X_7_ and glial fibrillary acidic protein (GFAP) in the hippocampus of rats with diabetic neuropathic pain (DNP) and major depressive disorder (MDD) (model). Data are displayed as the means ± standard error of the mean. **p < 0.01 vs. control group; ^##^ *p* < 0.01 vs. model group. Scale bars, 200 µm.

At the same time, the immunoreactivity of P2X_7_ in the DRGs, spinal cord, and hippocampus was detected using double-immunofluorescence labeling for GFAP and P2X_7_. GFAP marks satellite glial cells (SGCs). We found that P2X_7_ and GFAP coexpression in these regions were higher in model than in control rats (*p* < 0.05). However, the treatment with DHM or P2X_7_ shRNA reversed these changes (**Figures 5**–**7**). The up-regulation of GFAP in the DRGs, spinal cord, and hippocampus of rats in the model group suggested the activation of SGCs after a nervous-injury stimulus. Moreover, we found that P2X_7_ receptor was expressed by SGCs in all three regions. Therefore, DHM might decrease the expression of P2X_7_ receptor in these regions in rats with comorbid DNP and MDD.

### Effects of DHM on TNF-α and IL-1β Serum Levels in Rats With Comorbid DNP and MDD

**Figure 8:**
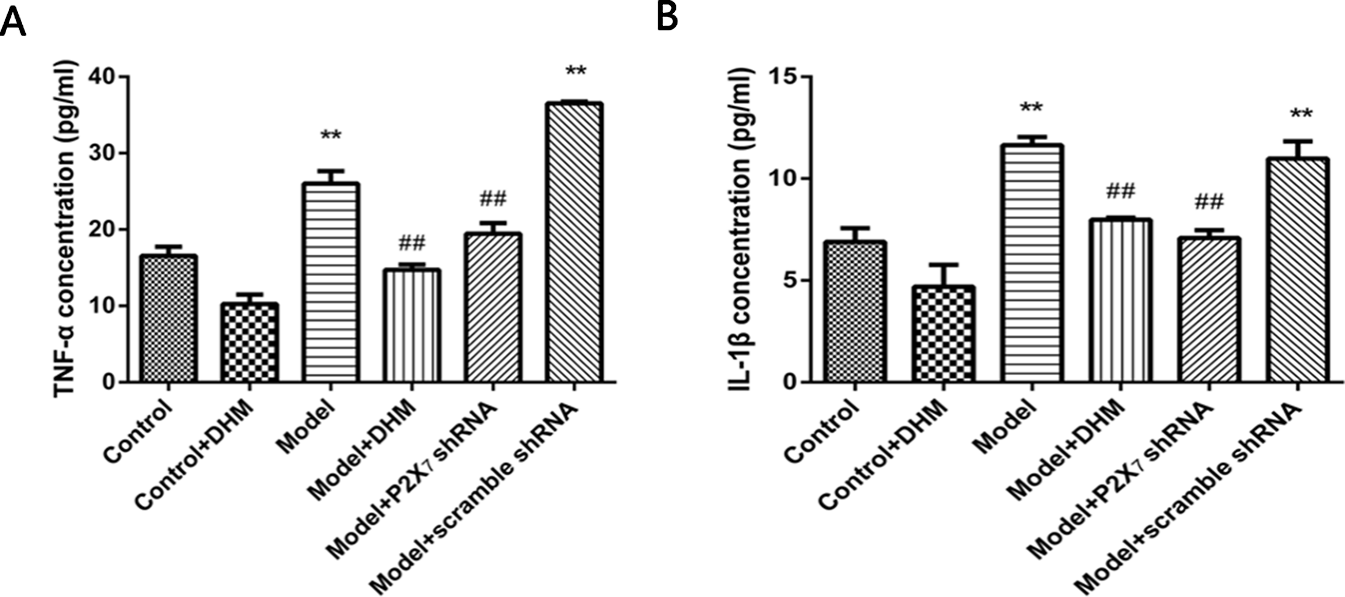
Effects of dihydromyricetin (DHM) on the expression of tumor necrosis factor α (TNF-α) **(A)** and interleukin 1ß (IL-1ß) **(B)** in the serum of rats with diabetic neuropathic pain (DNP) and major depressive disorder (MDD) (model). Data are displayed as the means ± standard error of the mean. **p < 0.01 vs. control group; ^##^ *p* < 0.01 vs. model group.

ELISA was used to detect the levels of TNF-α and IL-1β in the serum of rats with comorbid DNP and MDD. In both cases, the levels were higher in the model than in the control group (*p* < 0.05), while treatment with DHM or P2X_7_ receptor shRNA significantly reduced them (**Figure 8**).

### Effects of DHM on ERK1/2 Phosphorylation in DRGs, Spinal Cord, and Hippocampus of Rats With Comorbid DNP and MDD

**Figure 9:**
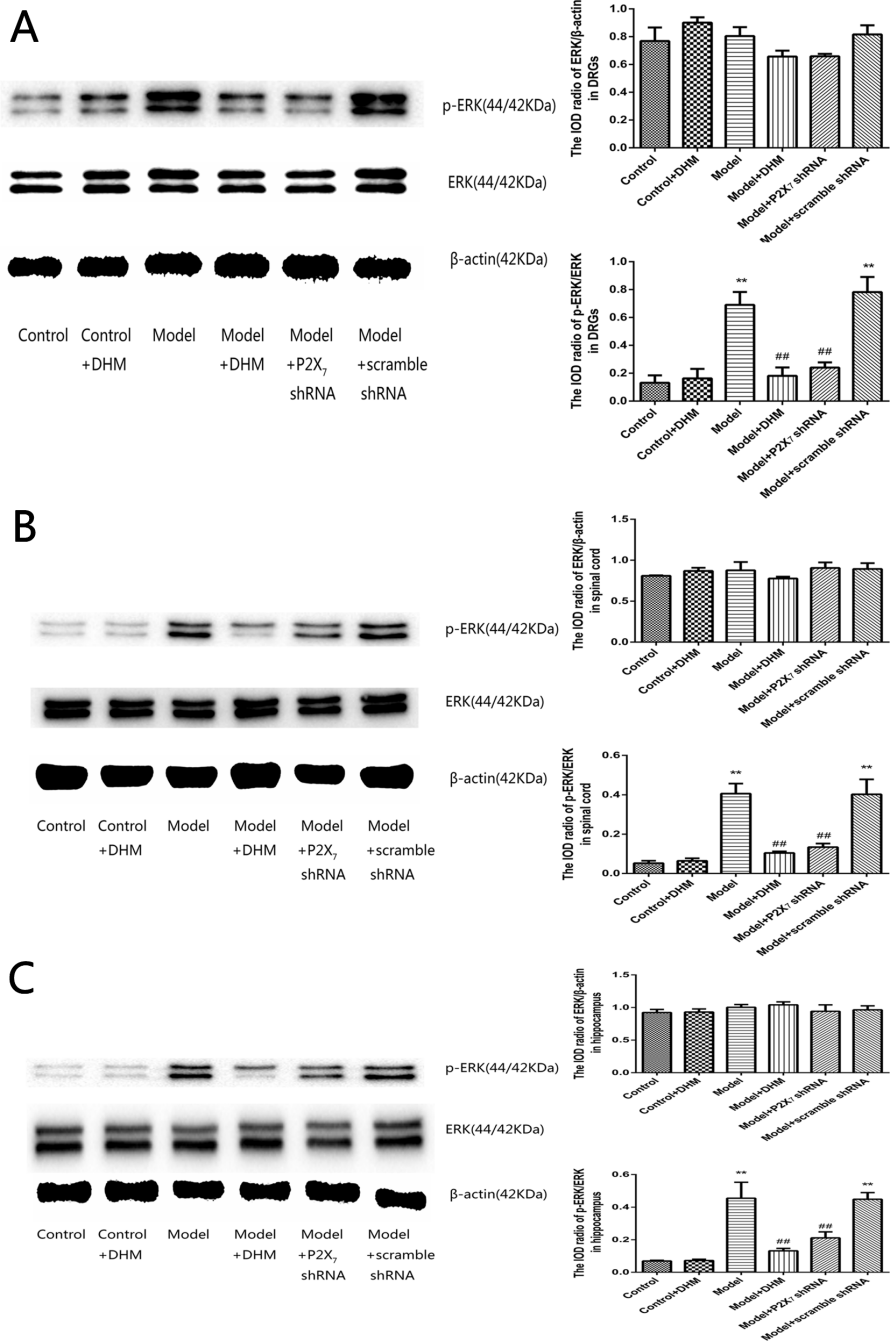
Effects of dihydromyricetin (DHM) on the levels of phosphorylated extracellular signal–regulated kinase 1/2 (ERK1/2) in the dorsal root ganglia (DRGs; **A**), spinal cord **(B)**, and hippocampus **(C)** of rats with diabetic neuropathic pain (DNP) and major depressive disorder (MDD) (model). Data are displayed as the means ± standard error of the mean. **p < 0.01 vs. control group; ^##^ *p* < 0.01 vs. model group.

The detection of p-ERK was performed by Western blotting. The levels of p-ERK in the DRGs, spinal cord, and hippocampus were higher in the model than in the control group (*p* < 0.05). However, treatment with DHM or P2X_7_ receptor shRNA reversed this increase (**Figure 9**).

### Discussion

Diabetes mellitus leads to a series of complications, including DNP and MDD, both of which are risk factors for the development of diabetes mellitus and mutually affect each other (23–27). The comorbid presence of DNP and MDD is difficult to clinically manage and reduces the patients’ quality of life to a greater extent than the individual presence of either DNP or MDD does (26, 28). In this study, we established a comorbid DNP and MDD rat model by administering a high-glucose and high-fat diet, STZ, and CUS (19). The successful generation of this model was confirmed by significantly reduced TWL values, MWT values, SP values, and OFT distances, as well as significantly increased ITs in the FST.

P2X_7_ receptor is a member of the purinergic receptor family (11, 29) and plays a specific role in nociceptive signaling during chronic pain states (15). Studies have also shown that P2X_7_ receptor is associated with anxiety and/or depressive symptoms, and P2X_7_ receptor antagonists may exert an antidepressant effect (18, 30). Our previous work showed that P2X_7_ receptors in the DRGs are involved in pain transmission in DNP and that down-regulation of P2X_7_ receptor expression relieves DNP (31). Moreover, we found that P2X_7_ receptor expression in the hippocampus was significantly higher in rats with comorbid DNP and MDD than in control rats (19). In the present study, we confirmed that comorbid DNP and MDD are associated with increased expression of P2X_7_ receptor and the corresponding mRNA in the hippocampus, DRGs, and spinal cord.

DHM is a natural flavone extracted from *A. grossedentata* and has many pharmacological effects, including antioxidative, anti-inflammatory, and neuroprotective effects (32, 33). Homology modeling and molecular docking analysis can predict ligand binding to a protein’s active sites. Molecular docking computations with AutoDock Vina (34) showed that DHM is capable of high-affinity binding to P2X_7_ receptor. In this study, we found that treatment with DHM or P2X_7_ receptor shRNA relieves neuropathic pain and depressive behaviors in rats with comorbid DNP and MDD and counteracts their elevated P2X_7_ receptor expression levels. The antidepressant-like effect of DHM in our study is consistent with the findings of Ren et al. (21). In addition, DHM treatment also relieved DNP symptoms in our study. Therefore, DHM might be an effective medication for the comorbid disease, and P2X_7_ receptor might be a key target for treatment.

Based on our findings, we hypothesize that patients with comorbid DNP and MDD have elevated P2X_7_ receptor expression in the DRGs, spinal cord, and hippocampus, and treatment with DHM or P2X_7_ receptor shRNA to inhibit P2X_7_ receptor expression can alleviate the symptoms of comorbid DNP and MDD.

In the nervous system, P2X_7_ receptor is expressed by glial cells, including satellite glia, astrocytes, and microglia (35–37). In this study, double-immunofluorescence labeling showed the coexpression of P2X_7_ receptor and GFAP (a marker of satellite glia or astrocytes) (38) in the DRGs, spinal cord, and hippocampus; this was more enhanced in the model than in the control group, while DHM or P2X_7_ receptor shRNA treatment reversed these changes. These results indicate that SGCs in the aforementioned regions are activated during DNP and MDD comorbidity, consistently with the observed increase in P2X_7_ receptor expression. Glial cells participate in the immune response by activating P2X_7_ receptor, thus enabling the release of a large number of proinflammatory cytokines (39). A significant feature of P2X_7_ receptor activation is the release of proinflammatory cytokines, which in turn affects cell activity (13, 39). P2X_7_ receptor plays an important role in the neuroinflammatory pathway, and its antagonists have been proposed as feasible drugs for the treatment of neuroinflammatory diseases (40, 41). Moreover, diabetes mellitus leads to increased expression of proinflammatory cytokines, such as TNF-α and IL-1β (42, 43), and increased inflammation is also considered to be involved in the pathogenesis of depressive symptoms in type 2 diabetes mellitus (44). Our team previously found that TNF-α and IL-1β serum levels were significantly higher in rats with DNP than in control rats (45, 46). In this study, we used ELISA and showed that the serum levels of IL-1β and TNF-α were significantly higher in model than in control rats but significantly decreased after DHM or P2X_7_ receptor shRNA treatment. We hypothesize that P2X_7_ receptor is activated in glial cells in the DRGs, spinal cord, and hippocampus during DNP and MDD comorbidity, thus stimulating the production and release of TNF-α and IL-1β to promote the pathogenesis of the two conditions.

Mitogen-activated protein kinase is involved in MDD and peripheral nerve injury-induced neuropathic pain, which mainly includes three pathways: ERK, p38 kinase, and c-Jun N-terminal kinase (47, 48). Many studies have confirmed that ERK pathway is closely associated with DNP and MDD (49, 50) and that its activation is involved in P2X-mediated pain and depressive symptoms (10, 51). Our previous experiments also revealed that the phosphorylation of ERK1/2 in the hippocampus is noticeably higher in rats with DNP and MDD than in control rats (19). In this study, we did not detect any significant changes among groups in the total ERK1/2 levels, but the levels of ERK1/2 phosphorylation in the DRGs, spinal cord, and hippocampus were significantly higher in the model than in the control group, indicating that the activation of this pathway might mediate pain and depression signal transduction in the case of DNP and MDD comorbidity. The fact that DHM or P2X_7_ receptor shRNA reduced the levels of p-ERK1/2 in the model group indicates that P2X_7_ receptor is associated with ERK pathway activation, involved in DNP and MDD. We found that DHM and P2X_7_ receptor shRNA treatments had similar effects. Therefore, DHM may inhibit the activation of ERK1/2 pathway by decreasing the expression of P2X_7_ receptor, reducing the secretion of inflammatory cytokines in peripheral glial cells, restraining pain and depression transduction, and thereby alleviating the symptoms of pain and depression.

One limitation of our study is that we used a rat model. Due to the complexity of DNP and MDD, rat models cannot completely mimic the human symptoms. Moreover, multiple pathways and receptors are involved in the progression of these disorders, and there are drugs other than DHM that may also effectively treat these disorders, such as palmatine (19). Further mechanistic research into the interaction between DHM and the P2X_7_ receptor, including research with human subjects, is therefore required.

In conclusion, we showed that P2X_7_ receptors in the DRGs, spinal cord, and hippocampus participate in the transduction of DNP- and MDD-related signals. DHM decreases P2X_7_ receptor expression in rats with comordid DNP and MDD, down-regulates ERK1/2 pathway activation, and reduces the release of the inflammatory factors TNF-α and IL-1β. These effects ultimately alleviate DNP and depressive behaviors. We propose that DHM may be an effective new drug for treating patients with comorbid DNP and MDD.

## Author Contributions

SG conducted the experiments with assistance from YS, HG, LL, CY, XW, LH, and WX. SG, WX, and YG contributed to the experimental design, data analysis and interpretation, and writing.

## Funding

This study was supported by grants from the National Natural Science Foundation of China (nos. 81760152, 81460106, 81360136), Major Disciplines of Academic and Technical Leaders Project of Jiangxi Province (no. 20142BCB22001).

## Conflict of Interest

The authors declare that the research was conducted in the absence of any commercial or financial relationships that could be construed as a potential conflict of interest.
